# Clinical and Genetic Factors Associated with Progression of Geographic Atrophy Lesions in Age-Related Macular Degeneration

**DOI:** 10.1371/journal.pone.0126636

**Published:** 2015-05-11

**Authors:** Felix Grassmann, Monika Fleckenstein, Emily Y. Chew, Tobias Strunz, Steffen Schmitz-Valckenberg, Arno P. Göbel, Michael L. Klein, Rinki Ratnapriya, Anand Swaroop, Frank G. Holz, Bernhard H. F. Weber

**Affiliations:** 1 Institute of Human Genetics, University of Regensburg, Regensburg, D-93053, Germany; 2 Department of Ophthalmology, University of Bonn, Bonn, D-53127, Germany; 3 National Eye Institute, National Institutes of Health, Bethesda, MD 20892–1204, United States of America; 4 Macular Degeneration Center, Casey Eye Institute, Oregon Health & Science University, and Devers Eye Institute, Portland, Oregon 97239, United States of America; Schepens Eye Research Institute/Massachusetts Eye and Ear, Department of Ophthalmology, Harvard Medical School, Boston, MA, UNITED STATES

## Abstract

Worldwide, age-related macular degeneration (AMD) is a serious threat to vision loss in individuals over 50 years of age with a pooled prevalence of approximately 9%. For 2020, the number of people afflicted with this condition is estimated to reach 200 million. While AMD lesions presenting as geographic atrophy (GA) show high inter-individual variability, only little is known about prognostic factors. Here, we aimed to elucidate the contribution of clinical, demographic and genetic factors on GA progression. Analyzing the currently largest dataset on GA lesion growth (N = 388), our findings suggest a significant and independent contribution of three factors on GA lesion growth including at least two genetic factors (ARMS2_rs10490924 [P < 0.00088] and C3_rs2230199 [P < 0.00015]) as well as one clinical component (presence of GA in the fellow eye [P < 0.00023]). These correlations jointly explain up to 7.2% of the observed inter-individual variance in GA lesion progression and should be considered in strategy planning of interventional clinical trials aimed at evaluating novel treatment options in advanced GA due to AMD.

## Introduction

Age-related macular degeneration (AMD) is a common cause of blindness in Western societies with an estimated prevalence for all forms of the disease to reach almost 8.7% and prevalence rates higher in Europeans than Asians or Africans [1]. Well-founded estimates project the worldwide number of people with AMD in 2020 to 196 million and in 2040 to 288 million [1]. In the near future, this will dramatically increase the individual as well as the socioeconomic burden of the disease.

AMD can progress in a succession of stages from an early to an intermediate and finally to a late form, where atrophic and neovascular subtypes are distinguished [2]. The early form is characterized by abnormalities at the level of the retinal pigment epithelium (RPE) and depositions of extracellular material located predominantly between Bruch’s membrane and the RPE [3]. As this material accumulates, it becomes recognized clinically as individual drusen deposits. When drusen progress in size to greater than 125 microns in diameter, they are designated as large drusen and the eye is classified as having intermediate AMD. Drusen may become confluent and be associated with RPE hyper- and hypopigmentation. The presence of large drusen is a strong indicator of increased risk to develop a late form of the disease [4] which can manifest as geographic atrophy (GA), involving a gradual degeneration and disappearance of RPE, photoreceptor cells and the choriocapillaris layer of the choroid in the central retina. Another form of late stage manifestation is the exudative or neovascular phenotype, which is accompanied by choroidal neovascularisation (CNV) with subpigmentepithelial, subretinal and/or intraretinal extracellular fluid accumulation evolving to retinal scarring if left untreated [5]. Both late stage disease manifestations can exist at the same time in the same eye or mixed with neovascular AMD in one eye and GA in the fellow eye. While CNV development may be associated with rapid functional impairment, GA typically progresses slowly and eventually may extend beyond the macular area of the retina [5]. While intravitreal administration of anti-vascular endothelial growth factor agents are beneficial for the treatment of neovascular AMD [6], there is no proven therapy for GA.

The proportion of GA in late stage AMD is approximately 35–40% [7,8]. While the overall incidence of the neovascular form is more frequent, GA occurs more common in individuals over 85 years of age [8]. This further emphasizes the impact of GA on ageing populations, and underscores the need of effective treatment to prevent, slow or cure the disease.

GA lesions usually expand with an average growth rate of about 1.3 to 2.6 mm² per year [9–12] and may ultimately result in severe central vision loss [13]. While meta-analysis of genome-wide association studies for advanced stage AMD has identified at least 19 loci [14] and biological pathways underlying AMD are slowly getting recognized [15], limited information is available about the influence of genetic, demographic and clinical factors on GA growth. One study reported a significant contribution of a common, AMD risk associated haplotype in 10q26 (*ARMS2/HTRA1* locus) on GA progression as measured by lesion size [10], although this correlation was not replicated in two subsequent studies of similar sample size [16,17]. This inconsistency can be ascribed to a number of specifics in the respective studies, e.g. related to imaging (color fundus photographs vs fundus autofluorescence), correction for initial lesion size [18] or different summarization of obtained growth rates [19]. Also, the effect of the presence of GA in the fellow eye was found to be a significant modulator of GA growth [12].

Design and evaluation of state-of-the-art clinical trials in GA require knowledge on factors that contribute to the progression of atrophic lesions. To validate previous findings [10,12] and to identify novel factors correlated with GA lesion growth, we analyzed the currently largest dataset on GA and GA lesion growth by combining two available studies: (i) the Fundus Autofluorescence in Age-related Macular Degeneration Study (FAM) [20], a multicenter study conducted in Germany and (ii) the Age-Related Eye Disease Study (AREDS) conducted in the United States [9]. We validated earlier findings for variant ARMS2_rs10490924 and for the presence of bilateral GA. We also expanded the analysis and searched for novel genetic and demographic factors correlated with GA lesion growth. Taken together, our data provide evidence for significant correlations between GA lesion growth and ARMS2_rs10490924, C3_rs2230199 and the presence of GA in the fellow eye, respectively. These correlations are independent of each other and jointly explain up to 7.2% of the observed inter-individual variance of GA growth.

## Materials and Methods

### Ethics Statement

The study followed the tenets of the Declaration of Helsinki and was approved by the local Ethics Review Board at the University of Bonn (ID: 082/04) and the NIH (IRB operates under FWA00005897 and the IRB Blue Panel, IRB00005894). Informed written consent was obtained from each patient after explanation of the nature and possible consequences of the study.

### Study characteristics

The study characteristics are summarized in Table 1. For screening (FAM—discovery) and initial validation (FAM—replication), we included a total of 201 eyes from 134 patients from the Fundus Autofluorescence in Age-Related Macular Degeneration (FAM) study (www.clinicaltrials.gov: NCT00393692) [16]. To validate the findings (AREDS—replication), we included 328 eyes from 254 patients from the Age-Related Eye Disease Study (AREDS) [10].

**Table 1:** Summary characteristics of participating study populations.

	FAM—discovery	FAM—replication	ARED replication	combined
Imaging technique	FAF	FAF	color fundus	mixed
Number of individuals	86	48	254	388
Mean follow-up time (S.D.) [years]	3.19 (1.97)	2.77 (1.66)	5.21 (3.00)	4.46 (2.85)
Mean interval between examinations (S.D.) [years]	1.37 (0.86)	1.56 (1.22)	1.13 (0.37)	1.24 (0.68)
Mean number of examinations (S.D.)	3.95 (2.86)	2.85 (1.61)	4.72 (2.83)	4.31 (2.78)
Mean age (S.D.) [years]	75.47 (7.37)	76.77 (5.90)	70.27 (5.07)	72.22 (6.36)
Mean growth [mm²/year] (S.D.)	1.62 (0.96)	1.34 (0.92)	1.55 (1.74)	1.54 (1.51)
Mean √growth [mm/year] (S.D.)	0.28 (0.14)	0.28 (0.14)	0.32 (0.30)	0.30 (0.25)
Mean ln (√growth [mm/year]) (S.D.)	-1.40 (0.60)	-1.43 (0.58)	-1.54 (1.03)	-1.50 (0.91)
Patients with bilateral GA [%]	67.4	64.6	29.1	40.72
Mean initial size (S.D.) [mm²]	6.53 (4.4)	5.03 (5.02)	2.96 (4.24)	4.01 (4.62)
Fraction male [%]	39.5	31.3	43.3	41.0

FAF = fundus autofluorescence.

### Classification of geographic atrophy

From the FAM-study, eyes with central (within a 500 μm radius of the foveal center) and non-central GA were included into the analysis and were classified as ‘GA’ eyes. In general, GA is funduscopically defined as one or more well-circumscribed, usually more or less circular patch of partial or complete depigmentation of the RPE, typically with exposure of underlying large choroidal blood vessels [9]. GA due to AMD is further defined as sharply demarcated lesion with clearly reduced FAF of an extend of ≥ 0.05 mm² (approximately 178μm in diameter) that does not correspond to exudative retinal changes (e.g. bleeding, exudates, fibrous scar) in an eye with funduscopically visible soft drusen and/or retinal pigment abnormalities consistent with AMD [12]

For the AREDS Study, GA associated with AMD was defined on stereoscopic color fundus photographs as sharply circumscribed areas of RPE depigmentation occurring in the macular area, generally considered to be circular in shape, with obvious visualization of the underlying choroidal blood vessels. The size must be as large as 1/8 disk diameter. Areas of RPE atrophy within or adjacent to fibrosis or other features of neovascularization is not considered GA. Central GA is defined as the involvement of the center of the fovea, which was determined by retinal vascular configuration and pigment change. The digitized images were evaluated for GA area (mm^2^) using computerized planimetry.[10].

### Fundus autofluorescence (FAF) measurements and calculation of GA growth rate

In the FAM study, FAF was measured and the lesion area was determined as previously reported [16] while AREDS analyzed the area of GA by color fundus photographs [10]. To eliminate the dependency of growth rates on baseline lesion size measurements, the individual area measurement was square root transformed (√area [mm]) [18]. We calculated lesion growth per examination interval by dividing the root transformed area by the time between examination points (in [years]), yielding a linear growth rate of the lesion (in [mm/year]). In case a growth rate was negative due to measurement imprecision, the growth rate for this interval was set at zero. The resulting growth rates per examination interval (√growth [mm/year]) were summarized for each individual by computing the mean of all growth rates for one individual. If data were available for both eyes of a patient, the mean of all computed growth rates from both eyes per individual were used. To generate normal distributed data and to reduce the bias from outliers, the growth rates per individual were log transformed with the natural logarithm (ln). This resulted in a single log-square-root growth rate variable per individual (ln(√growth [mm/year])), which was used for all subsequent analyses.

### Clinical and demographic variables

The age at first examination (in [years]), the gender and lesion sizes in one eye (unilateral GA) or both eyes (bilateral GA) at the last examination as well as the mean follow-up time of our patients and the mean interval between examinations (in [years], Table 1) were recorded. Additionally, we report the mean number of examinations each individual received (minimum: 2, maximum: 21) in order to exclude a confounding effect of the number of examinations on GA growth (Table 1).

### Genotyping and genetic risk score (GRS)

Genotyping was performed as described [10,21]. Briefly, genomic DNA was extracted from peripheral blood leukocytes by established methods. Genotyping was performed by TaqMan SNP genotyping (Applied Biosystems, Foster City, USA) or by PCR followed by restriction enzyme digestion (New England Biolabs, Ipswich, USA) and subsequent restriction fragment length analysis (RFLP).The resulting genotypes were coded as the number of AMD risk increasing alleles (0, 1 or 2), i.e. alleles which are more frequent in cases than in controls (S1 Table) [21]. These variants were used to compute the genetic risk score according to Grassmann *et al*. 2012 with weights obtained from the parsimonious model based on 10 SNPs (S1 Table).

### Statistical analyses and visualization

To visualize the raw data, we computed means and 95% confidence intervals of growth rates in different subgroups and used the *ggplot* function from the *ggplot2* [22] package in R [23] for drawing jitterplots. Linear regression was done to evaluate correlations between clinical and genetic variables with GA growth and computed P-values and confidence intervals as implemented in R. Furthermore, obtained P-values were adjusted by a conservative Bonferroni correction multiplying the P-values with the number of (independent) tests performed. Since we conducted the study in a three stage setup, we subsequently combined the obtained slopes and standard errors from the three independent studies by using the function *rma* from the packages *metafor* [24] and conducted the meta-analysis assuming a random effects model. This approach also allowed an assessment of heterogeneity between the estimates from each study.

## Results

### Study design

Overall, we investigated the effect of five clinical/demographic variables as well as ten genetic factors on GA lesion growth in 529 eyes from 388 individuals in a three stage study design (Table 1). Each area measurement was root transformed to eliminate the dependence of the growth rate on the initial lesion size [18]. A single growth rate per individual was calculated by taking the means of all calculated growth rates for each individual. In a first step, we aimed to replicated the findings from an earlier study for risk haplotype on 10q26 (*ARMS2/HTRA1* locus) [10]. This study showed that the risk increasing allele at ARMS2_rs10490924 increased GA lesion growth rate. We then searched for significant correlations between GA growth and novel clinical and genetic factors in a discovery study including 86 randomly selected individuals (*FAM study—discovery*) and considered factors which showed a nominally significant correlation (P_raw_< 0.05) for further replication. Significant findings were replicated in two additional studies (*FAM study—replication* and *AREDS—replication*, N = 302 individuals) and a meta-analysis was conducted to combine the effect sizes and standard errors from each individual study assuming a random effects model. The final P-values were adjusted by a conservative Bonferroni correction assuming 16 independent statistical tests and corrected P-values (P_corrected_) below 0.05 were considered significant. Lastly, we fitted a multivariate linear regression model to evaluate the independence of significantly correlated factors.

### Influence of ARMS2_rs10490924 risk allele on GA growth

**Fig 1:**
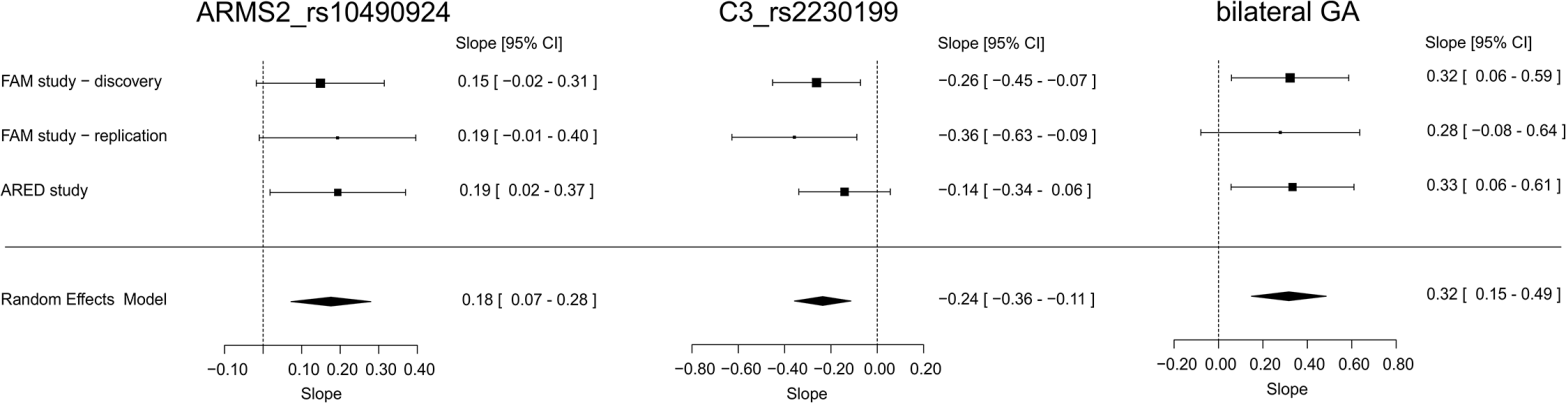
Forestplot representations of univariate linear regression models. Univariate linear regression models were fitted for variables ARMS2_rs10490924, C3_rs2230199 and bilateral GA for each study separately. Slope and standard errors obtained from the models of each study were combined by performing a meta-analysis assuming a random effects model. The combined estimates for slope and 95% confidence intervals (CI) were computed from the random effects model. In all analyses, no evidence was found for heterogeneity (P_heterogeneity_ > 0.05).

**Fig 2:**
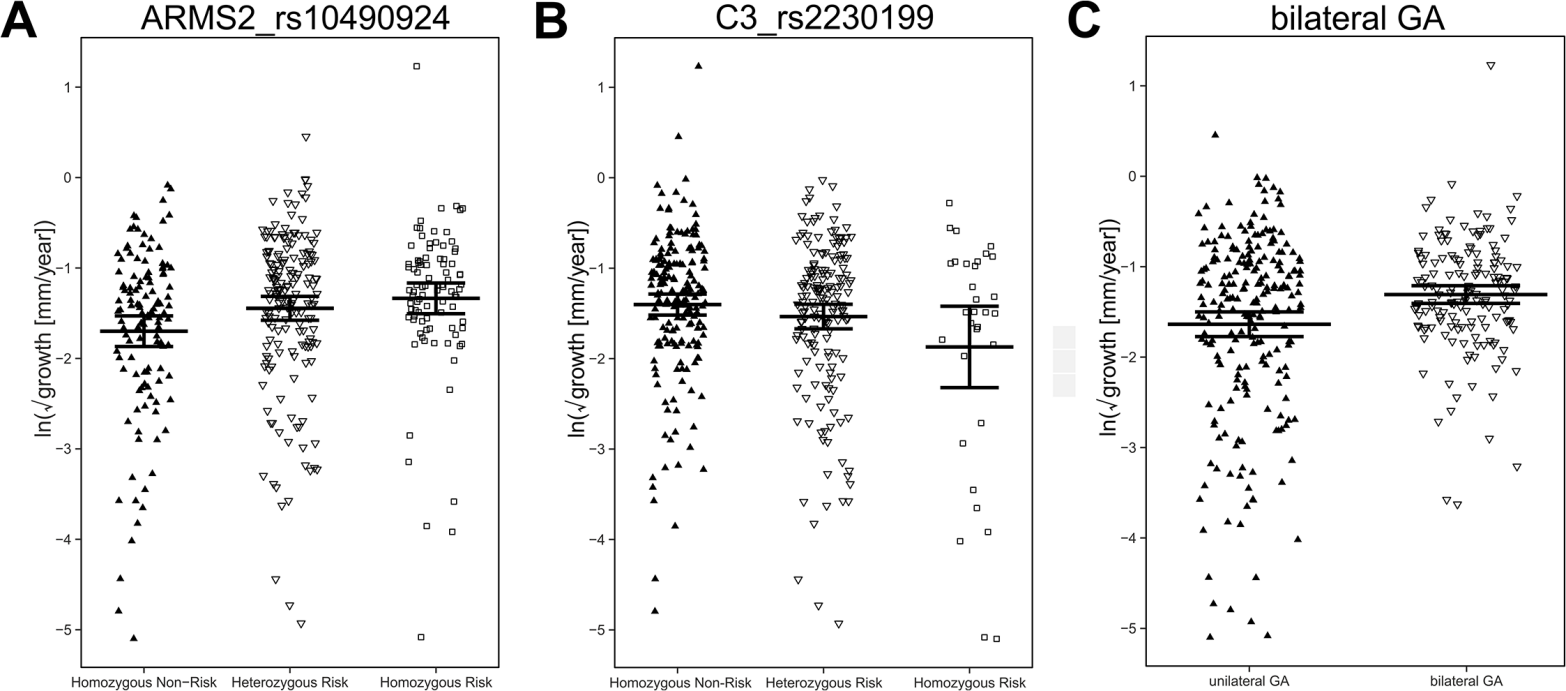
GA lesion growth rates for each individual in the combined study. The measured area of GA was square-root transformed. From the transformed area the growth rate was calculated per year in [mm/year]. Growth rates from each individual were then obtained by calculating the mean of all growth rates of the individual. If both eyes were affected, the mean of both eyes were calculated resulting in a single growth variable per individual. These individual growth rates were further transformed by the natural logarithm (ln) and were stratified either by (A) the genotype at ARMS2_rs10490924 or (B) the genotype at C3_rs2230199 or (C) the presence or absence of bilateral GA.

Klein *et al*. [10] found a significant contribution of the ARMS2_rs10490924 risk allele on GA lesion growth in 114 individuals from AREDS. We expanded these analyses by calculating the correlation of the number of risk alleles at this variant with GA growth in an extended AREDS panel of 254 patients (Table 2). We found a nominally significant correlation (P = 0.032) and a positive slope (0.194, 95% CI: 0.017–0.370) consistent with the previous study [10]. The findings were replicated in both FAM studies (*FAM—discovery* and *FAM—replication*) and the obtained slopes and standard errors were pooled using a random effects model. The combined slope was 0.176 (95CI: 0.072–0.280) with a raw P-value of 0.00088 (Table 2 and Fig 1). This correlation remained statistically significant after correction for multiple testing (P_corrected_ = 0.0141) and no evidence for heterogeneity between the studies was found (P_het_ = 0.919). The correlation was visualized for the combined study (N = 388) by stratifying the patients according to their genotype (homozygous non-risk, heterozygous risk and homozygous risk) and plotting the growth rate for each individual (Fig 2).

**Table 2:** Correlation between genetic, clinical and demographic factors and GA growth.

	FAM—discovery			FAM—replication			AREDS—replication			Combined (random effects model)				
	Effect size	95% CI ^b^	P ^c^	Effect size	95% CI ^b^	P ^c^	Effect size	95% CI ^b^	P ^c^	Effect size	95% CI ^b^	P ^c^	Pcorr^d^	Pheter ^e^
Gender	0.036	-0.228–0.300	0.788	-	-	-	-	-	-	-	-	-	-	-
Age [years]	0.001	-0.016–0.019	0.866	-	-	-	-	-	-	-	-	-	-	-
Initial size [mm²]	0.015	-0.015–0.044	0.325	-	-	-	-	-	-	-	-	-	-	-
Genetic Risk Score^a^	0.037	-0.057–0.132	0.434	-	-	-	-	-	-	-	-	-	-	-
CFH_rs1061170	-0.042	-0.233–0.148	0.660	-	-	-	-	-	-	-	-	-	-	-
CFH_rs6677604	-0.181	-0.551–0.189	0.334	-	-	-	-	-	-	-	-	-	-	-
CFH_rs800292	0.243	-0.048–0.534	0.100	-	-	-	-	-	-	-	-	-	-	-
C3_rs2230199	-0.262	-0.455–-0.070	0.008	-0.358	-0.635–-0.081	0.012	-0.141	-0.340–0.057	0.162	-0.24	-0.358–-0.114	1.50E-04	0.0024	0.4186
ARMS2_rs10490924^f^	0.149	-0.019–0.317	0.082	0.193	-0.016–0.402	0.069	0.194	0.017–0.370	0.032	0.176	0.072–0.280	8.80E-04	0.0141	0.919
CFB_rs438999	0.054	-0.452–0.561	0.832	-	-	-	-	-	-	-	-	-	-	-
CFB_rs4151667	0.113	-0.743–0.969	0.793	-	-	-	-	-	-	-	-	-	-	-
APOE_rs7412	-0.195	-0.500–0.111	0.209	-	-	-	-	-	-	-	-	-	-	-
APOE_rs429358	0.086	-0.216–0.388	0.574	-	-	-	-	-	-	-	-	-	-	-
CFI_rs2285714	-0.146	-0.354–0.062	0.167	-	-	-	-	-	-	-	-	-	-	-
bilateral GA	0.322	0.054–0.590	0.019	0.278	-0.09–0.647	0.135	0.333	0.055–0.611	0.019	0.317	0.148–0.485	2.30E-04	0.0037	0.9704
No. of exams	0.006	-0.051–0.039	0.796	-	-	-	-	-	-	-	-	-	-	-

^a^ GRS computed with reduced (10 SNPs) set according to Grassmann et al. 2012

^b^ 95% confidence intervals

^c^ P value from linear regression model without covariates

^d^ P value adjusted for multiple testing (Bonferroni correction) assuming 16 tests performed

^e^ P value for evidence of heterogeneity from random effects model

^f^ previously been shown to influence GA growth. We used the FAM study to replicate this finding.

### Correlation of genetic factors with GA growth

To estimate the contribution of additional genetic factors to GA lesion growth, we genotyped nine common AMD associated variants at 5 loci in FAM—discovery (Table 2 and S1 Table). The frequencies of the variants in FAM—discovery were comparable to those observed in AMD cases in other studies which have been shown to significantly and independently influence the risk for AMD [21]. In addition, we calculated a genetic risk score (GRS) to summarize the genetic risk of the individual participants. The average GRS in the discovery study was 1.96 (S.D. = 1.37) and thus slightly higher than the observed values for GA patients in a previous study [21].

In the discovery study, neither the genetic risk score nor most single genetic variants analyzed revealed a significant influence on GA growth rate (P_raw_ > 0.05, Table 2) with the exception of variant C3_rs2230199 (P_raw_< 0.05). Interestingly, the risk increasing allele (C) at C3_rs2230199 reduced the growth rate with a slope of -0.262 (95% CI: -0.455–-0.070). Thus, individuals who have an increased risk to develop AMD due to the risk increasing allele at C3_rs2230199 show a reduced rate of GA lesion growth when compared to individuals who do not carry risk increasing alleles. To further validate this finding, we replicated this correlation in *FAM—replication* and *AREDS—replication*. We found similar negative slopes in both replication cohorts and pooled the findings in a mixed effects model (Fig 1). The random effects model had a slope of -0.236 (95% CI: -0.358–-0.114) and demonstrated a highly significant correlation of the number of C3_rs2230199 risk alleles with GA growth (P_raw_ = 1.5x10^-4^), which remained statistically significant after adjustment for multiple testing (P_corrected_ = 0.0024). Additionally, we found no significant evidence for heterogeneity between the studies (P_het_ = 0.4186).

### Correlation of clinical and demographic factors with GA growth

After root transforming the measured area prior to the calculation of the growth rates, a significant correlation between the initial lesion size (in [mm²]) or the root transformed initial lesion size (in [mm]) and the growth rate of the lesion was not observed (Table 2). We also found no significant correlation to gender, age or the number of examinations to determine the GA growth rate. However, we found a strong correlation between the presence of GA in the fellow eye (bilateral GA) and GA growth (Table 2), in agreement with a previous report [12]. The findings indicate a significant increase in GA growth in cases where GA is present in both eyes of an individual. The slope of the regression model was estimated to be 0.322 (95% CI: 0.054–0.590) in the discovery study. To validate this finding, the effect of bilateral GA was investigated in the two replication cohorts. In each replication study, we found similar effect sizes in the same direction as observed in the discovery study. The findings were summarized in a meta-analysis of slopes and standard errors obtained from the individual studies (Fig 1). The random effects model showed a highly significant correlation (slope: 0.317, 95% CI: 0.148–0.485) of the presence of bilateral GA with GA growth (P_raw_ = 0.00023), which remained significant after adjustment for multiple testing (P_corrected_ = 0.0037, Table 2). Again, no significant evidence for heterogeneity between the studies was found (P_het_ = 0.9704). The growth rates for each individual were stratified according to the presence or absence of bilateral GA and were visualized in a jitterplot (Fig 2).

### Multivariate linear regression models

After correlating risk alleles at ARMS2_rs10490924 and presence of bilateral GA with GA lesion growth and identification of a novel genetic variant (C3_rs2230199) in the univariate regression analysis, we further evaluated the possibility that one of these factors influences or confounds the correlation with the other factors. We therefore fitted a multivariate linear regression model for each of the three studies and all three studies combined with the three identified factors in the same model (Table 3). No significant evidence for a confounding effect of these factors on the correlation of the other factors was found (P_corrected_ < 0.05). The adjusted slopes in the multivariate analyses differed on average by 0.048 (S.D. = 0.086) for ARMS2_rs10490924, 0.0946 (S.D. = 0.0913) for C3_rs2230199 and 0.034 (S.D. = 0.027) for bilateral GA when compared to the slopes in the univariate analysis. The combined regression model showed a highly significant fit (P_raw_ = 5.83x10^-8^) and an adjusted R² of 0.072, thus explaining up to 7.2% of the variation in the GA growth rate.

**Table 3:** Multivariate linear regression analysis of factors significantly correlated to GA growth.

	ARMS2_rs10490924	C3_rs2230199	bilateral GA	P ^a^
FAM—discovery	0.131 (-0.033–0.295)	-0.239 (-0.429–-0.049)	0.341 (0.084–0.598)	0.0014
FAM—replication	0.200 (-0.008–0.407)	-0.401 (-0.708–-0.094)	0.244 (-0.097–0.585)	0.0089
ARED—replication	0.205 (0.031–0.380)	-0.168 (-0.363–0.028)	0.362 (0.087–0.639)	0.0039
All combined ^b^	0.174 (0.072–0.276)	-0.232 (-0.355–-0.109)	0.326 (0.163–0.488)	5.83e-08

^a^ P value of linear regression model vs. null model

^b^ combined effect sizes were estimated from random effects model (meta-analysis).

## Discussion

Here, we aimed to further elucidate the contribution of genetic as well as clinical and demographic factors on the rate of AMD GA lesion enlargement. We extended previous efforts [10,16,17] and now provide data on the largest available dataset on GA lesion progression so far. If both eyes were affected, the previous studies either included only one eye at random [10,17] or pooled the data for both eyes [16]. Here, we chose to combine the growth rates observed for the two eyes of an individual to a single variable under the assumption that a germline genetic variant should influence lesion growth in a similar fashion in both eyes. And indeed, we and others report a high degree of concordance between GA growth rate in the two affected eyes of the patient [9,11,12,25,26]. Of noted, the ARED study reveals a lower occurrence of bilateral GA which can be explained mainly by two findings. Firstly, the ascertainment strategy was different for AREDS and FAM. While the ARED study specifically recruited patients with unilateral late stage AMD at baseline, the FAM study protocol included both uni- and bilateral patients. Secondly, we observed a significantly lower mean age in the ARED study compared to the FAM study. As individuals with unilateral GA are much more likely to progress to advanced stages in the fellow eye than individuals without late AMD manifestations [27,28], we expect the number of bilateral GA cases to increase in the ARED study over time.

A number of earlier studies have not reported a significant influence of genetic factors on GA lesion growth after adjustment for multiple testing [10,16,17]. Nevertheless, the correlation for C3_rs2230199 and ARMS2_rs10490924 presented in this report, was also suggested in a previous study which was based on a subsample of the present patient cohort [16]. However, the correlation in the previous work was not statistically significant after adjustment for multiple testing. Furthermore, in the FAM study several eyes were excluded from the current analysis which exhibited FAF phenotypes reminiscent of monogenetic diseases (e.g. GPS-FAF pattern [29] or CACD-FAF phenotypes [30]). In addition, we note that previous studies usually did not account for the large influence of the initial size of the lesion on the rate of progression possibly confounding the analyses. By root transforming the measured GA areas, we eliminated this problem. Furthermore, the present study included more than twice the number of participants than each single previous study and, thus, had a much higher power to detect significant correlations.

Our data confirm a significant correlation between GA lesion size and the number of risk alleles at ARMS2_rs10490924, a variant which represents the AMD risk haplotype at the *ARMS2*/*HTRA1* locus [31]. As the functional gene at this locus has not yet been identified [32], it is inappropriate to speculate about the mechanisms involved by which this locus may contribute to both the development as well as the progression of the disease.

At the C3 locus, the risk increasing allele at C3_rs2230199 reduced the growth rate of GA lesions. As the risk increasing allele at C3 results in reduced levels of CFH binding and thus in overly active complement [33], the observed statistical correlation between lesion size and C3 risk variant is inverse to findings for AMD risk and C3 risk variant and thus rather counter-intuitive. Complement activation is generally thought to be associated with increased inflammation due to priming [34] and activation of microglia [35] and as such would be expected to be associated with faster disease progression. On the other hand, several studies demonstrated low levels of inflammation to be beneficial: (i) *C3* knockout mice (C3^-/-^) implicated the complement system in neurogenesis [36] by showing that increased levels of complement activation promoted neurogenesis in healthy and diseased neuronal tissue; (ii) in the presence of immune cells low levels of complement activation revealed neuroprotective properties [37] and (iii) properdin, the only positive regulator of the alternative complement pathway, was found to be a protective factor in inflammatory diseases, thus shedding new light on the complement activation in neurodegenerative and inflammatory diseases [38]. Taking these findings into account, active neurogenesis and neuroprotection due to increased complement activation could counteract neurodegenerative activities in GA and thus could reduce lesion growth [39,40].

Our study replicated an earlier report on the influence of disease status of the fellow eye on GA lesion growth [12]. We demonstrate that (i) the slope, and thus the effect size, for bilateral GA is virtually the same in all cohorts analyzed in this study and (ii) that this correlation is independent of C3_rs2230199 and ARMS2_rs10490924. The ARMS2_rs10490924 variant increases the risk of progression from early to late AMD [41] as well as the growth rate of GA lesions. Thus, this variant may explain the increased growth in bilateral GA patients by (i) promoting the onset of GA in the fellow eye and (ii) increasing the growth rate of the lesion. However, in the multivariate model, both variables (*ARMS2* variant and bilateral GA) show an independent correlation with GA growth. Additionally, no significant association of ARMS_rs10490924 is detected with the presence of bilateral GA in the combined dataset (OR_Allele_: 0.810, 95% CI: 0.606–1.078) or in any individual study analyzed. Furthermore, the risk increasing alleles at C3_rs2230199 are independently correlated with a decreased progression rate. This finding argues strongly against a confounding effect of risk increasing genetic factors on the increased growth rate observed in bilateral GA patients, specifically, as risk increasing alleles at C3_rs2230199 reduce GA lesion growth. As no significant association is observed between C3_rs2230199 risk alleles and the presence of bilateral GA (OR_Allele =_ 1.087, 95% CI: 0.788–1.498), other factors must influence the onset of GA in the fellow eye and explain the increased growth rate in bilateral GA.

In summary, our findings reveal a significant and independent contribution of at least two genetic factors and the presence of GA in the fellow eye to GA lesion growth. Our studies have an impact on design and evaluation of future clinical trials aimed at testing novel treatment approaches for GA. These factors can greatly confound the outcome, particularly when GA growth rate is the main outcome parameter. As the area and growth rate of GA lesions do not necessarily correlate with visual acuity due to the frequently observed phenomenon of foveal sparing over a long period of time [13], it is difficult to draw any conclusion from our data on how long the patient’s vision can be retained. Future studies will be needed to evaluate the impact of genetic and clinical factors on visual perception.

## Supporting Information

S1 Table Allele frequencies of evaluated genetic variants in this study. (DOC)
