# Supplementary material for: Clinical and Genetic Factors Associated with Progression of Geographic Atrophy Lesions in Age-Related Macular Degeneration
Source: PLoS One. 2015 May 11;10(5):e0126636. doi: 10.1371/journal.pone.0126636 (PMC4427438; doi:10.1371/journal.pone.0126636)
Supplement: S1 Table — (DOC) [file pone.0126636.s001.doc]

|  |  |  |  | **FAM - discovery** | **FAM - replication** | **AREDS - replication** | **combined** |
| --- | --- | --- | --- | --- | --- | --- | --- |
| **Variant** | **Functional consequence** | **Risk allele*** | **Weight**** | **Allele frequency** | **Allele frequency** | **Allele frequency** | **Allele frequency** |
| CFH_rs1061170 | CFH_Y402H | C | 0.629 | 0.669 | - | - | - |
| CFH_rs6677604 | proxy: ΔCFHR1/3 | G | 0.702 | 0.930 | - | - | - |
| CFH_rs800292 | CFH_I62V | G | 0.932 | 0.913 | - | - | - |
| C3_rs2230199 | C3_R102G | C | 0.377 | 0.326 | 0.229 | 0.305 | 0.300 |
| ARMS2_rs10490924 | ARMS2_A69S | T | 1.301 | 0.483 | 0.479 | 0.451 | 0.461 |
| CFB_rs438999 | proxy: CFB_R32Q | T | 1.026 | 0.965 | - | - | - |
| CFB_rs4151667 | CFB_L9H | T | 1.406 | 0.988 | - | - | - |
| APOE_rs7412 | APOE_R158C | T | 0.397 | 0.093 | - | - | - |
| APOE_rs429358 | APOE_C112R | T | 0.335 | 0.901 | - | - | - |
| CFI_rs2285714 | - | T | 0.169 | 0.413 | - | - | - |

**S1 Table.** Allele frequencies of evaluated genetic variants in this study

* Risk allele refers to the risk increasing allele, i.e. the allele that is more frequent in cases than in controls

** Weights were obtained from the multiple logistic regression model in Grassmann et al. 2012 and used to calculate

the genetic risk score (GRS)
